# Apalutamide, enzalutamide, and darolutamide for non-metastatic castration-resistant prostate cancer: a systematic review and network meta-analysis

**DOI:** 10.1007/s10147-020-01777-9

**Published:** 2020-09-14

**Authors:** Keiichiro Mori, Hadi Mostafaei, Benjamin Pradere, Reza Sari Motlagh, Fahad Quhal, Ekaterina Laukhtina, Victor M. Schuettfort, Mohammad Abufaraj, Pierre I. Karakiewicz, Takahiro Kimura, Shin Egawa, Shahrokh F. Shariat

**Affiliations:** 1 Department of Urology, Medical University of Vienna, Währinger Gürtel 18-20, 1090 Vienna, Austria; 2 Department of Urology, The Jikei University School of Medicine, Tokyo, Japan; 3 Research Center for Evidence Based Medicine, Tabriz University of Medical Sciences, Tabriz, Iran; 4 Deaprtment of Urology, PRES Centre Val de Loire, CHRU Tours, France, Université François Rabelais de Tours, Tours, France; 5 Department of Urology, King Fahad Specialist Hospital, Dammam, Saudi Arabia; 6 Institute for Urology and Reproductive Health, Sechenov University, Moscow, Russia; 7 Department of Urology, University Medical Center Hamburg-Eppendorf, Hamburg, Germany; 8 Division of Urology, Department of Special Surgery, The University of Jordan, Amman, Jordan; 9 Cancer Prognostics and Health Outcomes Unit, University of Montreal Health Centre, Montreal, Canada; 10 Department of Urology, Weill Cornell Medical College, New York, NY USA; 11 Department of Urology, University of Texas Southwestern, Dallas, TX USA; 12 Karl Landsteiner Institute of Urology and Andrology, Vienna, Austria; 13 Department of Urology, Second Faculty of Medicine, Charles University, Prague, Czech Republic; 14 European Association of Urology Research Foundation, Arnhem, Netherlands

**Keywords:** Non-metastatic castration-resistant prostate cancer, Network meta-analysis, Apalutamide, Darolutamide, Enzalutamide

## Abstract

Management of non-metastatic castration-resistant prostate cancer (nmCRPC) has undergone a paradigm shift with next-generation androgen receptor inhibitors. However, direct comparative data are not available to inform treatment decisions and/or guideline recommendations. Therefore, we performed network meta-analysis to indirectly compare the efficacy and safety of currently available treatments. Multiple databases were searched for articles published before June 2020. Studies that compared overall and/or metastasis-free and/or prostate-specific antigen (PSA) progression-free survival (OS/MFS/PSA-PFS) and/or adverse events (AEs) in nmCRPC patients were considered eligible. Three studies (*n* = 4117) met our eligibility criteria. Formal network meta-analyses were conducted. For MFS, apalutamide, darolutamide, and enzalutamide were significantly more effective than placebo, and apalutamide emerged as the best option (*P* score: 0.8809). Apalutamide [hazard ratio (HR): 0.85, 95% credible interval (CrI): 0.77–0.94] and enzalutamide (HR: 0.86, 95% CrI: 0.78–0.95) were both significantly more effective than darolutamide. For PSA-PFS, all three agents were statistically superior to placebo, and apalutamide emerged as the likely preferred option (*P* score: 1.000). Apalutamide (HR: 0.71, 95% CrI: 0.69–0.74) and enzalutamide (HR: 0.76, 95% CrI: 0.74–0.79) were both significantly more effective than darolutamide. For AEs (including all AEs, grade 3 or grade 4 AEs, grade 5 AEs, and discontinuation rates), darolutamide was the likely best option. Apalutamide and enzalutamide appear to be more efficacious agents for therapy of nmCRPC, while darolutamide appears to have the most favorable tolerability profile. These findings may facilitate individualized treatment strategies and inform future direct comparative trials.

**Electronic supplementary material:**

The online version of this article (10.1007/s10147-020-01777-9) contains supplementary material, which is available to authorized users.

## Introduction

Prostate cancer is the most common solid cancer and the second most common cause of cancer-related death in men [1]. Systemic therapy based on androgen deprivation is the standard primary treatment strategy in patients with advanced prostate cancer. Despite adequate therapy, the disease eventually progresses to castration-resistant prostate cancer (CRPC) [2]. While docetaxel has long been the only agent with level 1 evidence for improved overall survival (OS) in metastatic CRPC (mCRPC), the advent of novel drugs/treatments, such as enzalutamide, abiraterone acetate, cabazitaxel, sipuleucel-T, and radium-223 has revolutionized therapeutic strategies for mCRPC [3–10].

CRPC without any metastases on conventional imaging is classified as non-metastatic CRPC (nmCRPC); preventing or delaying progression to metastatic disease is an area of unmet clinical need among patients with nmCRPC [11]. Recently, the phase III PROSPER, SPARTAN, and ARAMIS trials conducted in patients with high risk nmCRPC demonstrated enzalutamide, apalutamide, or darolutamide to be associated with a significantly longer median metastasis-free survival (MFS) compared to placebo [12–14]. Based on these results, guidelines have recommended them in patients with nmCRPC with a prostate-specific antigen doubling time (PSADT) of less than 10 months [15].

However, the available data directly comparing the effectiveness and safety of these agents are scarce to inform optimal treatment decisions and guideline recommendations. Moreover, long-term results of the PROSPER trial were recently reported [16]. Therefore, we conducted a systematic review of all clinical trials assessing treatment with next-generation androgen receptor inhibitors for nmCRPC using placebo as the control arm, and performed network meta-analyses to indirectly compare the efficacy and safety of these agents.

## Methods

### Search strategy

The systematic review and network meta-analysis of randomized controlled trials (RCTs) comparing systemic therapies for nmCRPC (with placebo as the control arm) were conducted according to the preferred reporting items for systematic reviews and meta-analyses (PRISMA) extension statement for network meta-analysis [17]. The PubMed, Web of Science, and Scopus databases were searched to identify reports published until June 2020 on systemic therapy for nmCRPC. The following keywords were used in our search strategy: (prostate carcinoma OR prostate cancer OR prostatic carcinoma OR prostatic cancer) AND (non-metastatic OR no metastatic OR M0) AND (castration resistant OR castration refractory OR hormone refractory OR hormone resistant) AND (Randomized). The primary outcome of interest was MFS, and the secondary outcomes were PSA-PFS, OS, and adverse events (AEs). Initial screening was performed independently by two investigators based on the titles and abstracts of the article to identify ineligible reports. Reasons for exclusions were noted. Potentially relevant reports were subjected to a full-text review, and the relevance of the reports was confirmed after the data extraction process. Disagreements were resolved via consensus with the co-authors.

### Inclusion and exclusion criteria

Studies were included if they investigated nmCRPC patients (Patients) who had undergone systemic therapy (Intervention) compared with those treated with placebo (Comparison) to assess the differential effects on MFS, PSA-PFS, OS, and AEs (Outcome) in a randomized studies only. We excluded observational studies, reviews, letters, editorials, meeting abstracts, replies from authors, case reports, and articles not published in English. References of all papers included were scanned for additional studies of interest. Studies were included only if they involved patients who received placebo as the control arm.

### Data extraction

Two investigators independently extracted the following information from the included articles: first author’s name, publication year, period of patient recruitment, number of patients, treatment dosage, age, study design, oncologic outcomes, and AE outcomes. Subsequently, the hazard ratios (HR) and 95% confidence intervals (CI) associated with MFS, PSA-PFS and OS, and AE rate were retrieved. HRs were extracted from cox analyses. All discrepancies regarding data extraction were resolved by consensus with the co-authors.

### Risk of bias assessment

The “risk-of-bias” (RoB) evaluation of each study was assessed according to The Cochrane Collaboration’s tool for assessing risk of bias [18]. This tool assesses selection bias (random sequence generation and allocation concealment), performance bias, detection bias, attrition bias, reporting bias, and other sources of bias (Supplementary Figure. 1). The RoB of each study was assessed independently by two authors. Disagreements were resolved by consultation with the co-authors.

### Statistical analyses

MFS was defined as the time from randomization to the first detection of distant metastasis on imaging or death. For each outcome, we conducted network meta-analysis using random and fixed effect models with a Bayesian approach for the direct and indirect treatment comparisons with placebo as the common comparator arm [19, 20]. In the assessment for MFS, PSA-PFS and OS, contrast-based analyses were applied with estimated differences in the log HR and the standard error calculated from the published HR and CI[21]. The relative treatment effects were presented as HR and 95% credible interval (CrI) [19]. With regard to MFS, subgroup analyses were conducted among: PSADT ≤ 6 M and PSADT > 6 M. For the assessment of the AEs, arm-based analyses were performed to estimate odds ratios (OR) of the AEs (and 95% CrI) from the available raw data presented in the selected manuscripts [19]. We also estimated the relative ranking of the different treatments for each outcome by using the *P* score, which can be considered a frequentist analog to the surface under the cumulative ranking curves [22, 23]. Network plots were utilized to illustrate the connectivity of the treatment networks in terms of MFS, PSA-PFS, OS, and AEs. Heterogeneity was assessed using I^2^ when more than one trial was available for a given comparison. All statistical analyses were performed using *R* 3.6.3 and Stata/MP 14.2 (Stata Corp., College Station, TX); statistical significance was set at *P* < 0.05.

## Results

### Study selection and characteristics

**Fig. 1:**
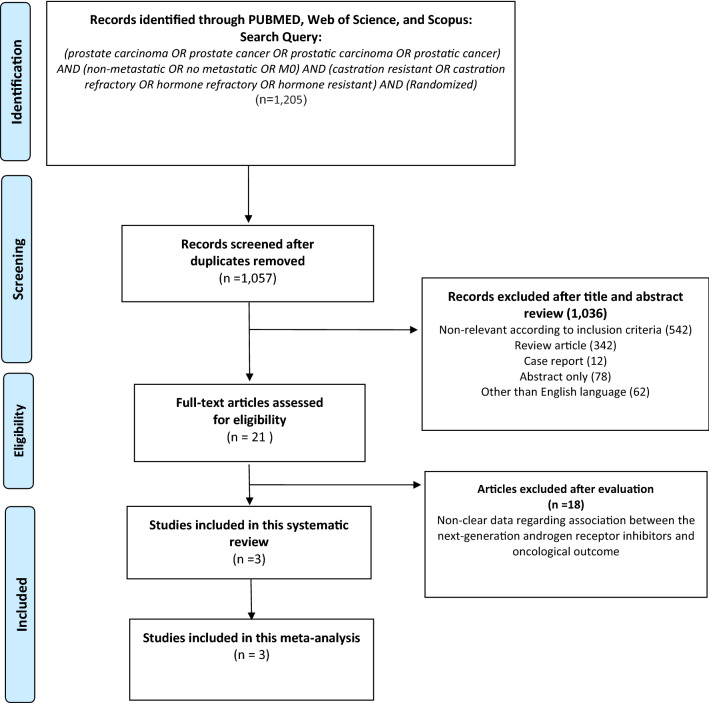
The Preferred Reporting Items for Systematic Reviews and Meta-analyses (PRISMA) flow chart, detailing the article selection process

Our initial search identified 1205 publications, and after the elimination of duplicates, a total of 1057 publications were available. A total of 1036 articles were excluded after screening the titles and abstracts, and a full-text review was performed for 21 articles (Fig. 1). Based on the selection criteria, we identified 3 articles comprising 4117 patients for the systematic review and network meta-analysis [12–14, 16, 24]. Extracted data from the three studies are outlined in Table 1. All these studies were published between 2018 and 2020 and included a total of 1423 patients (median age: 73–74 years, median PSADT: 3.6–4.7 months) treated with placebo and 2694 patients (median age: 74 years, median PSADT: 3.8–4.4 months) treated with a next-generation androgen receptor inhibitors.

**Table 1:** Study characteristics

Trial	PROSPER	SPARTAN	ARAMIS
Author	Hussain	Smith	Fizazi
Year	2018	2018	2019
Agents	Enzalutamide+ADT	Apalutamide+ADT	Darolutamide+ADT
Dosage	160mg	240mg	600mg
Control	Placebo+ADT	Placebo+ADT	Placebo+ADT
Inclusion criteria	M0N0CRPC, PSADT < 10 months, PSA >2 ng/ml	M0N0-N1CRPC, PSADT <10 months	M0N0-N1CRPC, PSADT < 10 months, PSA >2 ng/ml
Number	1401	1207	1509
Number (Treatment)	933	806	955
Number (Control)	468	401	554
Median Age (range)	74 (50−95) vs. 73 (53−92)	74 (48−94) vs. 74 (52−97)	74 (48−95) vs. 74 (50−92)
Median PSA at baseline (ng/ml)	11.1 vs. 10.2	7.78 vs. 7.96	9.0 vs. 9.7
Median PSADT (months)	3.8 vs. 3.6	4.4 vs. 4.5	4.4 vs. 4.7
Proportion of N1	0% vs. 0%	16.5% vs. 16.2%	17% vs. 29%
Metastasis free survival	36.6 vs. 14.7, HR 0.29 95% CI 0.24-0.35	40.5 vs. 16.2, HR 0.28 95% CI 0.23-0.35	40.4 vs. 18.4, HR 0.41 95% CI 0.34-0.5
PSA progression free survival	37.2 vs. 3.9, HR 0.07 95% CI 0.05-0.08	NR vs. 3.7, HR 0.06 95% CI 0.05-0.08	33.2 vs. 7.3, HR 0.13 95% CI 0.11-0.16
Overall survival	67 vs. 56.3, HR 0.73 95% CI 0.61-0.89	NR vs. NR, HR0.75 95% CI 0.59-0.96	NR vs. NR, HR 0.71 95% CI 0.5-0.99
Any grade AE rate	87% vs. 77%	96.5% vs. 93.2%	83.2% vs. 76.9%
Grade 3 or 4 AE rate	31% vs. 23%	24.8% vs. 23.1%	24.7% vs. 19.5%
Grade 5 AE rate	3% vs. 1%	1.2% vs. 0.3%	3.9% vs. 3.2%
Discontinuation rate	9% vs. 6%	10.6% vs. 7.0%	8.9% vs. 8.7%
Median follow up (months)	48	41	17.9

*ADT* androgen deprivation therapy, *CRPC* castration-resistant prostate cancer, *PSA* prostate-specific antigen, *PSADT* PSA doubling time, *NR* not reached, *HR* hazard ratio, *CI* confidential interval, *AE* adverse event

### Network meta-analysis

The networks of eligible comparisons are graphically represented in network plots in terms of MFS, PSA-PFS, OS, and AEs (Supplementary Figure. 2).

#### MFS

**Fig. 2:**
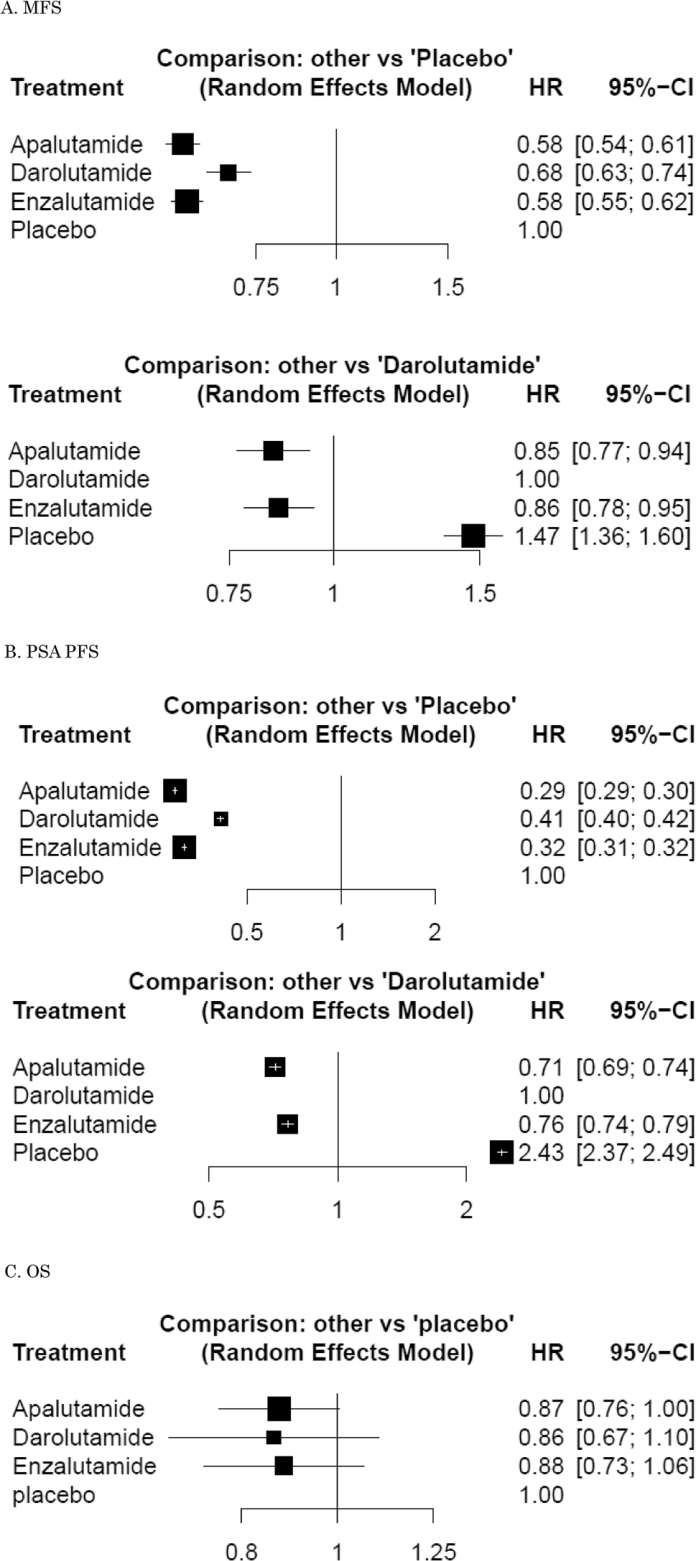
Forest plots showing the association of systemic therapy in non-metastatic castration-resistant prostate cancer. **a** metastasis-free survival (MFS), **b** prostate-specific antigen progression-free survival (PSA-PFS), **c** overall survival (OS)

A network meta-analysis of 3 different agents was conducted for the primary outcome of MFS. Compared with placebo, apalutamide, darolutamide, and enzalutamide resulted in a significantly improved MFS (HR: 0.58, 95% CrI: 0.54–0.61, HR: 0.68, 95% CrI: 0.63–0.74, and HR: 0.58, 95% CrI: 0.55–0.62, respectively) (Fig. 2a). Compared with darolutamide, apalutamide and enzalutamide resulted in a significantly improved MFS (HR: 0.85, 95% CrI: 0.77–0.94, and HR: 0.86, 95% CrI: 0.78–0.95, respectively). Based on analysis of the treatment ranking, apalutamide had the highest likelihood of providing the maximal MFS (*P* score: 0.8809), closely followed by enzalutamide (*P* score: 0.7852) (Table 2).

**Table 2:** Analysis of the treatment ranking

Treatment	*P* score (fixed)	*P* score (random)
Metastasis-free survival		
Apalutamide	0.8809	0.8809
Enzalutamide	0.7852	0.7852
Darolutamide	0.3339	0.3339
Placebo	0.000	0.000
PSA progression-free survival		
Apalutamide	1.0000	1.0000
Enzalutamide	0.6667	0.6667
Darolutamide	0.3333	0.3333
Placebo	0.0000	0.0000
Overall survival		
Apalutamide	0.6594	0.6594
Darolutamide	0.6589	0.6589
Enzalutamide	0.6024	0.6024
Placebo	0.0792	0.0792

#### PSA PFS

A network meta-analysis of three different agents was conducted for the secondary outcome of PSA-PFS. Compared with placebo, apalutamide, darolutamide, and enzalutamide resulted in a significantly improved PSA-PFS (HR: 0.29, 95% CrI: 0.29–0.30, HR: 0.41, 95% CrI: 0.40–0.42, and HR: 0.32, 95% CrI: 0.31–0.32, respectively) (Fig. 2b). Compared with darolutamide, apalutamide and enzalutamide resulted in a significantly improved PSA-PFS (HR: 0.71, 95% CrI: 0.69–0.74, and HR: 0.76, 95% CrI: 0.74–0.79, respectively). Based on analysis of the treatment ranking, apalutamide had the highest likelihood of providing the maximal PSA-PFS (*P* score: 1.0000) followed by enzalutamide (*P* score: 0.6667) (Table 2).

#### OS

A network meta-analysis of three different agents was conducted for the secondary outcome of OS. Compared with placebo, apalutamide, darolutamide, and enzalutamide did not result in a significantly improved OS (HR: 0.87, 95% CrI: 0.76–1.00, HR: 0.86, 95% CrI: 0.67–1.10, and HR: 0.88, 95% CrI: 0.73–1.06, respectively) (Fig. 2c). Based on analysis of the treatment ranking, apalutamide had the highest likelihood of providing the maximal OS (*P* score: 0.6594), closely followed by darolutamide and enzalutamide (*P* score: 0.6589 and 0.6024, respectively) (Table 2).

#### AEs

A network meta-analysis of three different agents was conducted for the various outcomes of AEs (including any AE, grade 3 or grade 4 AE, grade 5 AE, and discontinuation rates).

**Fig. 3:**
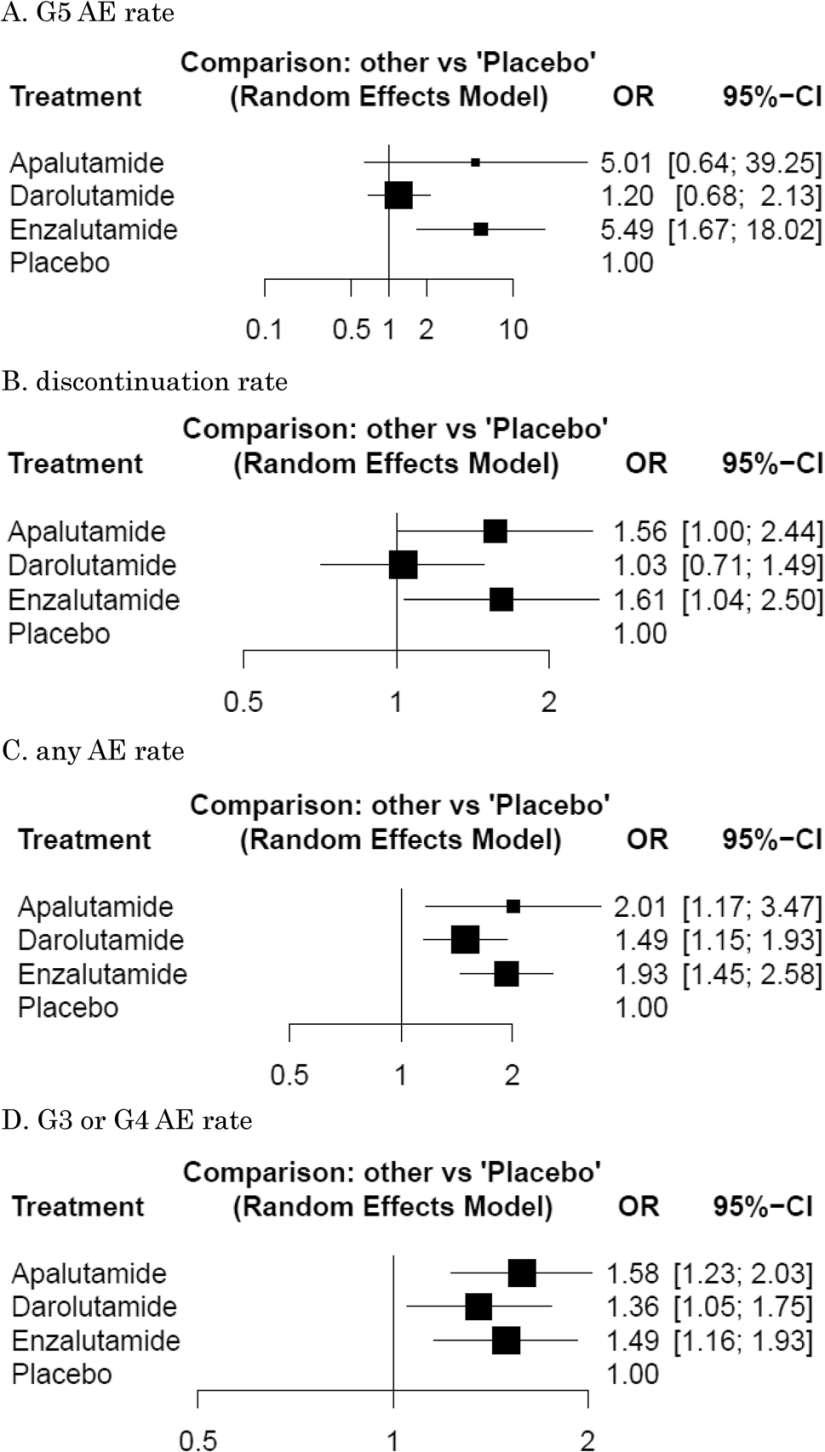
Forest plots showing the association of systemic therapy in non-metastatic castration-resistant prostate cancer. **a** grade (G) 5 adverse events (AE) rate, **b** discontinuation rate, **c** any AE rate, **d** G3 or G4 AE rate

Darolutamide caused similar number of Grade 5 AEs and discontinuation compared with placebo (OR: 1.20, 95% CrI: 0.68–2.13, and OR: 1.03, 95% CrI: 0.71–1.49, respectively). By contrast, apalutamide (OR: 5.01, 95% CrI: 0.64–39.25, and OR: 1.56, 95% CrI: 1.00–2.44, respectively) and enzalutamide (OR: 5.49, 95% CrI: 1.67–18.02 and OR: 1.61, 95% CrI: 1.04–2.50, respectively) were associated with a significantly higher likelihood of grade 5 and toxicity leading to discontinuation (Fig. 3a, b). All three drugs were associated with a significantly higher likelihood of toxicity regarding any AEs and grade 3 or grade 4 AEs (Fig. 3c, d). Based on analysis of the treatment ranking, it was highly likely that darolutamide had the lowest rate of all AE outcomes compared to both apalutamide and enzalutamide (Supplementary Table 1).

#### MFS (PSADT ≤ 6 M)

Compared with placebo, in patients with PSADT ≤ 6 M nmCRPC, apalutamide, darolutamide, and enzalutamide resulted in significantly improved MFS (HR: 0.58, 95% CrI: 0.55–0.62, HR: 0.68, 95% CrI: 0.62–0.75, and HR: 0.58, 95% CrI: 0.54–0.61, respectively) (Supplementary Figure. 3A). Compared with darolutamide, apalutamide and enzalutamide resulted in significantly improved MFS. Based on analysis of the treatment ranking, enzalutamide had the highest likelihood of providing the maximal MFS (*P* score: 0.8768), closely followed by apalutamide (*P* score: 0.7875) (Supplementary Table 2).

#### MFS (PSADT > 6 M)

Compared with placebo, in patients with PSADT > 6 M nmCRPC, apalutamide, darolutamide, and enzalutamide resulted in significantly improved MFS (HR: 0.59, 95% CrI: 0.52–0.68, HR: 0.66, 95% CrI: 0.57–0.76, and HR: 0.63, 95% CrI: 0.53–0.75, respectively) (Supplementary Figure. 3B). Based on analysis of the treatment ranking, apalutamide had the highest likelihood of providing the maximal MFS (*P* score: 0.8574) (Supplementary Table 2).

## Discussion

We conducted systematic review on systemic therapy agents that have been evaluated in placebo-controlled RCTs for patients with nmCRPC; we also performed network meta-analysis to indirectly compare the safety and efficacy of these therapies. This approach generated several important findings. First, only new androgen receptor inhibitors were adequately tested in this clinical disease space. Second, apalutamide emerged as the most likely best treatment option with regard to MFS and PSA PFS. Moreover, apalutamide and enzalutamide were significantly more effective than darolutamide with regards to MFS and PSA PFS. Conversely, third, darolutamide was the best-tolerated of all three agents evaluated causing a similar number of AEs compared to placebo with regard to grade 5 AE rate and discontinuation rate.

These developments are of particular interest, as previous network meta-analyses did not include recently reported data [25] [26]. Therefore, we included recently published data, such as updated results from the PROSPER and SPARTAN trials [16, 24]. In addition, this network meta-analysis included detailed AE outcomes. This is of greater relevance to clinical practice than the recent network meta-analyses [25, 26]. On these points, current paper may more readily facilitate individualized treatment selection.

Results from the ARAMIS, PROSPER, and SPARTAN trials showed positive effects of apalutamide, darolutamide, or enzalutamide, respectively, on the primary endpoint MFS [12–14]. Despite finding similar efficacy in MFS compared to placebo, investigators from the PROSPER trial included only patients without lymph node enlargement (N0) while the SPARTAN and ARAMIS trials included patients with lymph nodes up to 2 cm in diameter in the short axis (N1) below aortic bifurcation. Subgroup analyses in both trials indicated a potential benefit for apalutamide and darolutamide for patients with N1 status compared to N0 (HR 0.15 vs. 0.33 and HR 0.28 vs. 0.46, respectively) suggests that the efficacy of enzalutamide may have been unfairly estimated in comparison with the other agents. However, this possibility must be validated and investigated in more detail to obtain reliable data on the relevance of lymph node positivity and the therapeutic efficacy of these agents. Moreover, the inclusion criteria differed somewhat, with a minimum serum PSA of 2 ng/ml in ARAMIS and PROSPER, and no minimum in SPARTAN. Despite these differences, the present network meta-analysis indicated that apalutamide may be the most effective therapeutic option based on its benefits with regard to MFS and PSA PFS. Apalutamide is molecularly and mechanistically similar to enzalutamide, as it antagonizes the ligandbinding domain of androgen receptor (AR) with potent affinity, thereby preventing AR nuclear translocation; it also does not have agonistic effects in the presence of AR overexpression [27]. Apalutamide led to ≥ 50% tumor regression in eight of ten castrate immunodeficient mice harboring LNCaP/AR xenograft tumors, whereas bicalutamide led to ≥ 50% tumor regression in only one of the ten evaluated mice [27]. Notably, apalutamide also demonstrated greater in vivo activity in CRPC xenograft models [28], compared to enzalutamide, 2–4-times lower doses of apalutamide were needed to achieve stable, therapeutic plasma concentrations in a mouse model of human CRPC xenografts with approximately the same drug concentrations in the tumor; this suggests a higher therapeutic index for apalutamide with a greater scope for dose escalation [27].

Another important finding from this meta-analysis was that darolutamide was the best tolerated of all three evaluated agents. Darolutamide is a non-steroidal AR-antagonist with a molecular structure that is distinct from those of enzalutamide and apalutamide [29]. In general, darolutamide has advantage of fewer and less severe toxic effects than apalutamide and enzalutamide because of its low penetration of the blood–brain barrier and low-binding affinity for g-aminobutyric acid type A receptors based on preclinical studies [30, 31]. Indeed, darolutamide was associated with fewer central nervous system effects than either enzalutamide or apalutamide with respect to seizures (enzalutamide 11% and apalutamide 15.6% vs. darolutamide 4.2%), cognitive/mental impairment disorders (enzalutamide 5% and apalutamide 5.1% vs. darolutamide 0.4%) or dizziness (enzalutamide 10% and apalutamide 9.3% vs. darolutamide 4.5%). In addition, it is important to point out that patients with known central nerves system malignancies were included in the ARAMIS study, while they were excluded in PROSPER and SPARTAN. Moreover, both enzalutamide and apalutamide are strong CYP3A4 inducers and thus have potential for CYP-mediated drug-drug interactions such as decreased plasma exposure to warfarin via CYP inhibition [32]. By contrast, darolutamide is not a CYP-inhibitor and is, therefore, less likely to cause drug-drug interactions than enzalutamide or apalutamide [33].

Despite the comprehensive nature of this systematic review, there are some limitations that need to be considered when interpreting the results. First, although indirect treatment comparison analyses have been used and validated for comparing outcomes from RCTs, this approach falls short of a head-to-head treatment comparison. Thus, direct well designed comparative trials are required to validate the findings of this study. Second, this network meta-analysis was based on the reporting quality of the trials we reviewed and may have been affected by several types of biases, thus limiting the validity of the overall findings. Third, the patient characteristics may have differed significantly between the studies, thereby limiting the comparability of the trials evaluated. Indeed, as mentioned above, caution should be exercised in assessing the data on enzalutamide as the inclusion criteria for lymph node status differed from those applied in the other studies. Moreover, because of inherent limitations of published data, performing a meta-analysis of adjusted effect estimates proved to be impossible. Finally, differences in subsequent therapies received across the treatment arms in the trials evaluated may have potentially influenced the OS results. In addition, the OS data from some trials were still immature; thus, the study outcomes could change in their final analyses. In addition to these limitations, accurate distinction between non-metastatic and metastatic disease based solely on conventional imaging modalities could be problematic owing to their limited sensitivity. Interestingly, over 90% of men with nmCRPC determined according to conventional imaging were ultimately found to have metastases using prostate-specific membrane antigen positron emission tomography in retrospective series [34]. Hence, patients in the three trials analyzed in the present study should be considered to have low-volume metastatic disease rather than nmCRPC, and the primary endpoint of progression to metastases on conventional imaging seems less relevant as prostate-specific membrane antigen positron emission tomography imaging is increasingly used in clinical settings.

Despite these caveats, the current network meta-analysis suggests that apalutamide may be the most efficacious option for the treatment of nmCRPC while darolutamide seems the safest. Although this meta-analysis does not replace the need for head-to-head clinical trials of contemporary systemic therapies, this finding could help improve clinical decision-making until such direct comparative data become available.

## Conclusion

In this systematic review and network meta-analysis of first-line systemic therapies for patients with nmCRPC, based on an indirect comparison of data from placebo controlled phase 3 clinical trials, apalutamide was identified as having a higher likelihood of providing the maximum benefits in terms of MFS and PSA PFS. Darolutamide appeared to have the most favorable tolerability. These findings may provide guidance to patients and clinicians with regards to treatment decisions in conjunction with other aspects that drive personalized medicine strategies for nmCRPC.

## Electronic supplementary material

Below is the link to the electronic supplementary material.

Supplementary file1 (PDF 37 kb)

Supplementary file2 (PDF 31 kb)

Supplementary file3 (PDF 64 kb)

Supplementary file4 (DOCX 16 kb)

Supplementary file5 (DOCX 16 kb)
