# Supplementary material for: Apalutamide, enzalutamide, and darolutamide for non-metastatic castration-resistant prostate cancer: a systematic review and network meta-analysis
Source: Int J Clin Oncol. 2020 Sep 14;25(11):1892–900. doi: 10.1007/s10147-020-01777-9 (PMC7572325; doi:10.1007/s10147-020-01777-9)
Supplement: Supplementary file 1 — Supplementary file1 (PDF 37 kb) [file 10147_2020_1777_MOESM1_ESM.pdf]

Supplementary Figure 1

Risk of bias summary of the included studies for network meta-analysis

(A) Random sequence generation (selection bias); (B) allocation concealment (selection bias); (C) blinding of participants and personal (performance bias); (D) blinding of outcome assessment (detection bias); (E) incomplete outcome data (attrition bias); (F) selective reporting (reporting bias); (G) other bias. Green circles represent a low risk of bias and confounding, red circles represent a high risk of bias and confounding, and yellow circles represent an unclear risk of bias and confounding.

Author, year	*A*	*B*	*C*	*D*	*E*	*F*	*G*
Hussain 2018	+	+	+	+	−	+	+
Smith 2018	+	?	+	+	−	+	+
Fizazi 2019	+	+	+	+	−	+	+
