# Supplementary material for: Apalutamide, enzalutamide, and darolutamide for non-metastatic castration-resistant prostate cancer: a systematic review and network meta-analysis
Source: Int J Clin Oncol. 2020 Sep 14;25(11):1892–900. doi: 10.1007/s10147-020-01777-9 (PMC7572325; doi:10.1007/s10147-020-01777-9)

Supplementary Figure 2

Network plots showing the association of systemic therapy in non-metastatic castration-resistant prostate cancer

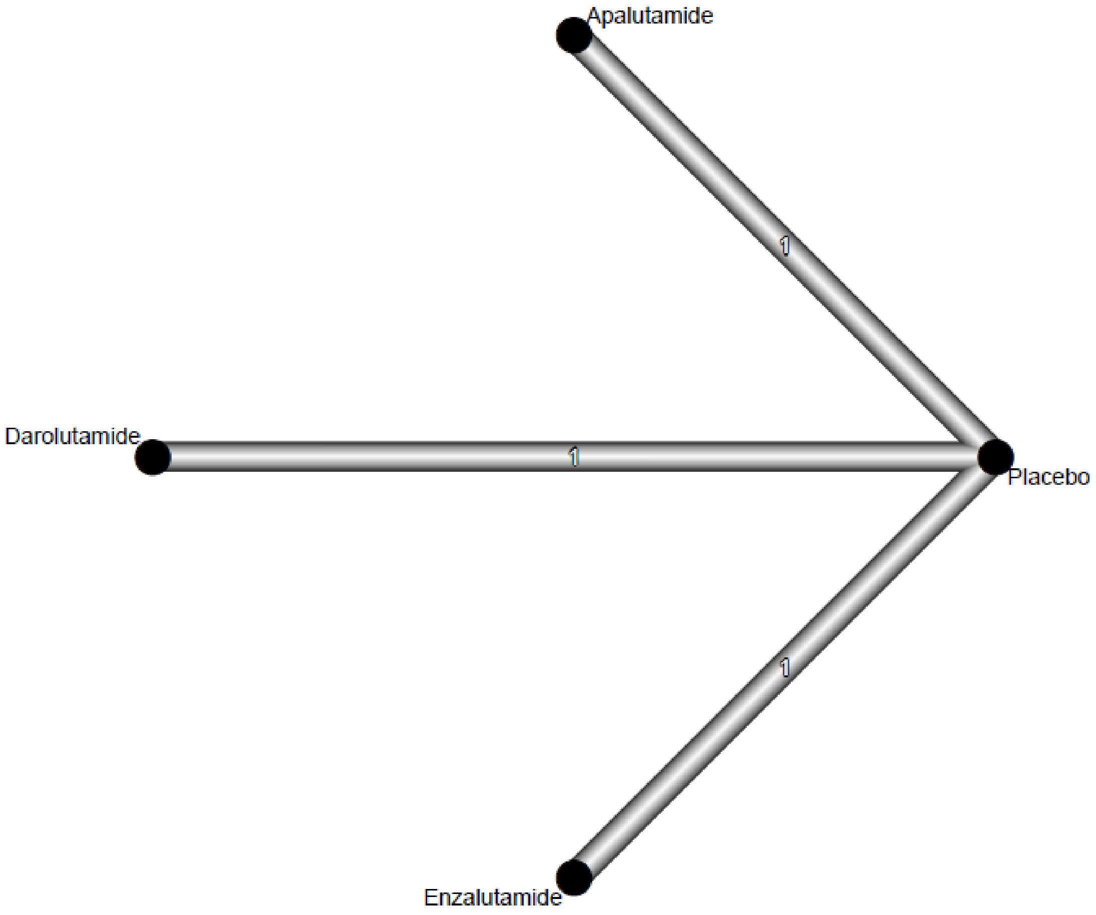

Supplement: Supplementary file 2 — Supplementary file2 (PDF 31 kb) [file 10147_2020_1777_MOESM2_ESM.pdf]
