# Supplementary material for: Apalutamide, enzalutamide, and darolutamide for non-metastatic castration-resistant prostate cancer: a systematic review and network meta-analysis
Source: Int J Clin Oncol. 2020 Sep 14;25(11):1892–900. doi: 10.1007/s10147-020-01777-9 (PMC7572325; doi:10.1007/s10147-020-01777-9)
Supplement: Supplementary file 4 — Supplementary file4 (DOCX 16 kb) [file 10147_2020_1777_MOESM4_ESM.docx]

Supplementary Table 1 Analysis of the treatment ranking

| Grade 5 adverse event rate | | |
| --- | --- | --- |
| Treatment | P score (fixed) | P score (random) |
| Placebo | 0.8899 | 0.8899 |
| Darolutamide | 0.7192 | 0.7192 |
| Apalutamide | 0.2294 | 0.2294 |
| Enzalutamide | 0.1615 | 0.1615 |
| Discontinuation rate | | |
| Treatment | P score (fixed) | P score (random) |
| Placebo | 0.8410 | 0.8410 |
| Darolutamide | 0.7640 | 0.7640 |
| Apalutamide | 0.2133 | 0.2133 |
| Enzalutamide | 0.1818 | 0.1818 |
| any adverse event rate | | |
| Treatment | P score (fixed) | P score (random) |
| Placebo | 0.9976 | 0.9976 |
| Darolutamide | 0.5805 | 0.5805 |
| Enzalutamide | 0.2164 | 0.2164 |
| Apalutamide | 0.2055 | 0.2055 |
|  |  |  |
| Grade 3 or grade 4 adverse event rate | | |
| Treatment | P score (fixed) | P score (random) |
| Placebo | 0.9963 | 0.9963 |
| Darolutamide | 0.5025 | 0.5025 |
| Enzalutamide | 0.3077 | 0.3077 |
| Apalutamide | 0.1934 | 0.1934 |
