# Supplementary material for: Apalutamide, enzalutamide, and darolutamide for non-metastatic castration-resistant prostate cancer: a systematic review and network meta-analysis
Source: Int J Clin Oncol. 2020 Sep 14;25(11):1892–900. doi: 10.1007/s10147-020-01777-9 (PMC7572325; doi:10.1007/s10147-020-01777-9)
Supplement: Supplementary file 5 — Supplementary file5 (DOCX 16 kb) [file 10147_2020_1777_MOESM5_ESM.docx]

Supplementary Table 2 Analysis of the treatment ranking

| Metastasis free survival (PSA doubling time ≦ 6 months) | | |
| --- | --- | --- |
| Treatment | P score (fixed) | P score (random) |
| Enzalutamide | 0.8768 | 0.8574 |
| Apalutamide | 0.7875 | 0.6319 |
| Darolutamide | 0.3357 | 0.5106 |
| Placebo | 0.0000 | 0.0000 |
| Metastasis free survival (PSA doubling time > 6 months) | | |
| Treatment | P score (fixed) | P score (random) |
| Apalutamide | 0.8574 | 0.8574 |
| Enzalutamide | 0.6319 | 0.6319 |
| Darolutamide | 0.5106 | 0.5106 |
| Placebo | 0.0000 | 0.0000 |
